# miR-5000-3p confers oxaliplatin resistance by targeting ubiquitin-specific peptidase 49 in colorectal cancer

**DOI:** 10.1038/s41420-021-00494-0

**Published:** 2021-06-01

**Authors:** Yan-yan Zhuang, Wa Zhong, Zhong-sheng Xia, Shu-zhen Lin, Man chung Chan, Ke Jiang, Wen-fei Li, Xin-yi Xu

**Affiliations:** Department of Gastroenterology, Sun Yat-sen Memorial Hospital, Sun Yat-sen University, Guangzhou, Guangdong 510000 China

**Keywords:** Colon cancer, Drug development

## Abstract

Colorectal cancer (CRC) is the most common form of gastrointestinal malignancies. A growing number of reports focusing on oxaliplatin (OXA) resistance in CRC treatment have revealed that drug resistance is an urgent issue in clinical applications, especially for finding effective therapeutic targets. Recently, microRNAs (miRNAs) are reported to play a critical role in tumor progressions and multi-drug resistance. The main aim of this study is to establish whether miR-5000-3p is an oncogene that is resistant to OXA and further confirm its underlying regulatory role in CRC. The OXA-associated gene expression dataset in CRC cells was downloaded from Gene Expression Omnibus (GEO) database. Statistical software R was used for significance analysis of differentially expressed genes (DEGs) between OXA-resistant (OR)-CRC cells and CRC cells, and results indicated ubiquitin-specific peptidase 49 (USP49) was upregulated in OR-CRC cells. Luciferase reporter assay showed that USP49 was verified to act as a downstream target gene of miR-5000-3p. From the results of TCGA database, miR-5000-3p expression was upregulated and USP49 was downregulated in patients with CRC. The function of miR-5000-3p was detected using MTT assay, wound healing, Transwell, and flow cytometry assays. Moreover, through in vitro and in vivo experiments, miR-5000-3p expression was confirmed to be upregulated in CRC cells or OR-CRC cells comparing to normal cell lines. Molecular mechanism assays revealed that USP49 binds to the miR-5000-3p promoter to increase the expression of miR-5000-3p, resulting in cancer cells sensitized to OXA. To sum up, these results suggest that miR-5000-3p may be a novel biomarker involved in drug-resistance progression of CRC. Moreover, the drug-resistance mechanism of miR-5000-3p/USP49 axis provides new treatment strategies for CRC in clinical trials.

## Introduction

Colorectal cancer (CRC) is one of the most common malignant tumors in the digestive system worldwide^1^. CRC patients are usually treated with surgery, radiation, chemotherapy, targeted drugs, or a combination of these therapies^2^. Despite advances in therapy, including surgery and chemotherapy, there are ~800,000 new patients diagnosed with CRC and almost 100,000 colon cancer patients die from CRC every year in China^3^. Due to colon cancer cells acquiring the features of multiple drug resistance (MDR), <50% of patients with colon cancer respond to the best regimen of combined chemotherapy^3,4^. Thus, exploring crucial molecules and biomarkers underlying CRC progression is of great importance for finding efficient strategies for CRC treatment.

miRNAs, a class of non-coding RNAs containing 21–23 nucleotides, have been shown to play an important role in cellular development, genomic imprinting, and regulating cellular functions^5^. Dysregulation of ncRNAs could regulate the progression of various cancers via the competing endogenous RNA (ceRNA) network. mRNAs act as tumor suppressors inhibiting tumor growth and as oncogenes promoting tumor growth by binding to the 3′-untranslated region (3′-UTR) of a non-coding region within target messenger RNAs (mRNAs) and leading to the mRNA destabilization and translational repression^6^. miR-5000-3p was found to be a potential miRNA biomarker of side population (SP) cells of CRC and provided a new specific target of the SP for the reversal of MDR of CRC^3^. Besides, knockdown of miR-5000-3p remarkably reduced cell proliferation and migration in laryngocarcinoma^7^. However, the role of miR-5000-3p in tumor resistance of CRC has not been reported, and how USP49 expression present in CRC is unclear.

Ubiquitin-specific peptidase 49 (USP49) is a deubiquitinase containing ubiquitin-specific protease domain and UBP-type zinc finger domain, which is involved in the modification of cellular proteins. Except for a report demonstrating that USP49 regulates co-transcriptional pre-mRNA splicing through histone H2B deubiquitination^8^. A previous study suggested depletion of USP49 increased pancreatic cancer cell proliferation in vitro, tumorigenesis in vivo, and chemoresistance, proposing that USP49 regulates tumorigenesis and chemoresistance in pancreatic cancer in an AKT-dependent manner^9^. USP49 was also reported to inhibit non-small cell lung cancer cell growth by suppressing PI3K/AKT signaling^10^. However, the cellular function of USP49 remains largely unknown. *USP49* was belonging to CRC-associated gene and found that can increase cell sensitivity to etoposide (Eto)-induced DNA damage and was suggested as a tumor suppressor during the development of CRC^11,12^. Therefore, we suspect that USP49 may serve a role in CRC progression or drug sensitivity, based on its low expression in the OXA-resistance CRC cells when compared to CRC cells for DEGs from GEO dataset in our study. By the prediction online from TargetScanHuman (http://www.targetscan.org/vert_72/), we found that miR-5000-3p could directly target with USP49 gene. However, it is not known whether USP49 and miR-5000-3p play a role in concert in CRC drug resistance.

Oxaliplatin (OXA) is a third-generation drug used in CRC first-line chemotherapy and has been used to treat stage III and stage IV colorectal cancer. Increasing reports indicated that OXA resistance in CRC treatment is an urgent problem to be solved in clinical applications. In the present study, we aim to clarify the role of miR-5000-3p/USP49 in the drug-resistance mechanism in an OXA-resistant CRC cell models and xenograft animal models.

## Materials and methods

### Bioinformatics analysis and identification of DEGs

The gene expression data (450 cases of CRC patients and 8 normal controls) and corresponding clinical information were downloaded from TCGA official website for the colon adenocarcinoma (COAD) patients. Boxplots were used to visualize expression differences for discrete variables. For GEO database searching, the microarrays related to oxaliplatin resistance in CRC were obtained from GEO database (www.ncbi.nlm.gov/geo). The search terms included ‘colorectal cancer’ or ‘oxaliplatin resistance’. Then, microarrays that contained mRNA probe were screened for further analysis.

### Human CRC tissue samples

We randomly collected 403 samples of primary CRC tissues were obtained from patients in Sun Yat-sen Memorial Hospital. All CRC patients were not complicated with other severe organic lesions, and lactating and pregnant women were not allowed to join the study. All samples were clinically and pathologically diagnosed by two experienced pathologists and were followed up regularly for up to 5 years. The detailed clinical information was shown in Table 1. Meanwhile, the corresponding adjacent 8 cases of non-tumorous colorectal tissues were collected. All tissues were stored at −80 °C until RNA extraction.

**Table 1:** Relationship between miR-5000-3p expression and tumor characteristics in patients with colorectal cancer.

Features	No. of patients	miR-5000-3p expression		*p* value
		Low	High	
All patients	403	201	202	
Age (years)				0.008**
<69	199	88	111	
≥69	204	117	87	
Gender				0.496
Male	215	113	102	
Female	188	92	96	
T Infiltrate				0.023*
T1	10	8	2	
T2	72	39	33	
T3	270	140	130	
T4	50	18	32	
Lymphatic metastasis (N)				0.400
0	253	133	120	
1	86	41	45	
2	64	31	33	
Metastasis (M)				0.666
0	292	150	142	
1	56	27	29	
Stage				0.290
1	72	41	31	
2	167	84	83	
3	97	47	50	
4	56	27	29	

*p < 0.05 and **p < 0.01.

### Cell lines culture and OXA-resistant cell establishment

Eight human CRC cell lines (HCT116, HCT8, HT29, LoVo, DLD-1, SW620, SW480, Caco-2), and a normal human intestinal epithelial cell line (HIEC-6) were purchased from Shanghai Cell Bank of Chinese Academy of Sciences (Shanghai, China). Cells were maintained in RPMI 1640 medium that was supplied with 10% fetal bovine serum (FBS; Invitrogen) and 1% penicillin-streptomycin (Sigma-Aldrich) at 37 °C in a 5% CO_2_ incubator.

Oxaliplatin-resistant (OR) CRC cells were established using HCT116 and LoVo cells following the protocol in a previous study^13^. Briefly, the drug-resistant CRC cell line was established by exposing CRC cells to the doses of 0–25 µM OXA in a dose-dependent manner. The IC_50_ (50% cell death) of OXA in HCT116 and LoVo cells was found to be the concentration of 15 µM which induces 90% cell death. The surviving cells were recovered to 80% in culture and then passaged in the same OXA concentration to increase the OXA dose. Herein, we selected the cell populations whose survival was under 3-fold to the OXA IC_50_ concentration (45 µM) and identified these cell populations as the OXA-resistant HCT116 and LoVo cell lines (OR-HCT116 and OR-LoVo cell lines).

### RT-qPCR analysis

Total RNA was separated from tissues and cells with the usage of TRIzol reagent (Invitrogen). RNA quality was examined by A260/A280 absorption. Briefly, cDNA was synthesized by using a TaqMan miRNA reverse transcription kit (Takara). The quantitative real-time PCR (RT-qPCR) was carried out with a SYBR Premix Ex Taq kit (TaKaRa) on an ABI 7500 RT-PCR system (Applied Biosystems, USA). Relative quantification was achieved by normalization to the amount of GAPDH mRNA or U6, and the relative expression levels of genes were calculated using 2^–ΔΔCT^ method.

### MTT assay

Cell viability was measured using the MTT [3-(4,5-dimethylthiazol-2-yl)-2,5-diphenyltetrazolium-bromide] assay (Sigma) after different treatments. In brief, cells (1 × 10^4^/per well) were seeded into 96-well plates and were treated with different concentrations of OXA or not treated. After 24 h of OXA exposure, 100 µ L of MTT (0.5 mg/mL) was added to the cells, followed by incubation for an additional 3 h. The blue MTT formazan crystals were then dissolved in 150 µ L of DMSO. Then, a ELISA microplate reader (Bio-Tek Instruments) at an absorbance of 570 nm was used to evaluate cell viability following: (treated − blank)/(untreated control − blank).

### Prediction of mRNA targets

Two established miRNA target prediction programs (starBase and TargetScanHuman 7.2) were used to predict the putative targets of miR-5000-3p.

### Cell transfections

The cells were 50–70% confluent at the time of transfection. HCT116, LoVo, OR-HCT116, and OR-LoVo cells were transfected with the plasmids of miR-5000-3p knockdown (inhibitor-1 and inhibitor-2), or overexpression (mimics-1 and mimics-2), scrambled miRNA vectors (mimics NC or inhibitor NC), and overexpression (OE) plasmids of USP49 (OE-USP49) and the corresponding NC (OE-NC), which were synthesized by Ribo-Bio Ltd. (Guangzhou, China). The cell transfections after 48 h were routinely conducted using Lipofectamine 2000 (Invitrogen) according to the manufacturer’s protocol.

### Wound-healing assay

A wound-healing assay was performed to assess cell migration after transfections. Briefly, cells were added to 6-well plates for culture reaching 80% confluence, and a sterile plastic micropipette tip was then used to scratch the cell monolayer. Subsequently, the cells were incubated under standard conditions for 24 h, the wound images were captured under an inverted microscope (Olympus, Tokyo, Japan). Cells were assessed in three random fields with the healing wound centered in the field at 0 and 24 h after removing the wall to quantify cell migration.

### Transwell assay

Cell invasive capacities were assessed by using Transwell chambers. A total of 2 × 10^4^ cells in the upper compartment of the chamber was added with serum-free medium, while culture medium containing an additional 10% FBS was added to the lower chamber. After 24 h of incubation at room temperature, the cells on the inner surface of the upper chamber were scraped with a cotton swab and the invaded cells in the lower chamber were fixed using ethyl alcohol and stained with 0.1% crystal violet for 15 min. After washed excessive dye with PBS and dried, the number of migratory cells was imaged and counted under an optical microscope (Olympus).

### Cell apoptosis assay

Cell apoptosis was assessed using Annexin V-APC/PI staining kit (BD Bioscience, USA). After 24 h of transfections, 1 × 10^4^ cells were incubated with 45 μM OXA for 48 h. Then, cells were collected, washed twice with PBS, and stained with Annexin V-APC for 15 min and propidium iodide (PI) for 5 min to label the cells. The percentage of apoptotic cells was measured by a FACSCanto flow cytometer (BD, USA).

### Luciferase reporter assay

The wild-type (WT) and mutant (MUT) binding sites of miR-5000-3p in the USP49 sequence 3′untranslated (UTR) region were subcloned into pmirGLO Dual-Luciferase vector to generate USP49-WT or USP49-MUT and then co-transfected with miR-5000-3p mimics or miR-NC into HCT116 and LoVo cells using Lipofectamine 2000 (Invitrogen). After 24 h transfection, the relative luciferase activities were normalized by Renilla luciferase activities and examined using a Dual-Luciferase reporter assay system (Promega).

### Subcutaneous tumor xenograft model

Four- to six-week-old male C57BL/6 nude mice were used for establishing xenograft models. All animal experiments were approved by the Institutional Animal Care and Use Committee of Sun Yat-sen University and were performed in accordance with the Guidelines for the Care and Use of Laboratory Animals from the National Institutes of Health, China. The animals were housed in an environment maintained at a temperature of 25 ± 2 °C and a 12 h dark-light cycle with the lights on from 8 am to 8 pm. Water was provided ad libitum throughout the experiment. Two cell lines (HCT116 cells and OR-HCT116 cells) at the concentration of 3 × 10^6^ were used to subcutaneously inject into the right flank of nude mice. When the tumors reached an average volume of 100 mm^3^, the mice were randomly divided into 10 groups (*n* = 5 mice in each group). According to the experimental design, intratumorally injection of miR-agomir NC, miR-5000-3p agomir, miR-antagomir NC, miR-5000-3p antagomir, or treatment with OXA was administered every 3 days with 3 mg/kg. The tumor size and weight were measured every 3 days. The tumor volume was calculated according to the following formula: volume = length × width^2^/2. Fifteen days after the OXA treatment, the mice were sacrificed according to ARRIVE guidelines. The tumor tissues were separated for IHC staining. All the tumor detections were performed independently by two pathologists who were blinded to the experimental group allocation during the experiment.

### Immunohistochemistry (IHC) staining

After the tumor’s tissues were paraffin-embedded, Ki-67 staining was performed to further evaluate tumor growth and the efficacy of OXA treatment. Briefly, tumors were removed, fixed with formalin, embedded with paraffin, and sectioned. IHC staining for Ki-67 was performed on 10-µm sections using the primary antibodies obtaining from Abcam (ab16667). The slides were then incubated with secondary antibodies and visualized using 3,3-diaminobenzidine tetrahydrochloride plus (DAB+) according to the manufacturer’s instructions. Counterstaining for nuclei was performed using hematoxylin and the staining intensity was assessed.

### Protein extraction and western blot analysis

Total proteins were extracted from cultured cells using RIPA Lysis Buffer (Beyotime) containing a protease inhibitor and phosphatase inhibitor (Roche). Protein concentrations were quantified using a BCA protein assay (Invitrogen). Protein samples were separated by SDS-PAGE and subsequently transferred onto nitrocellulose membranes (Millipore). Membranes were then sealed with 5% skim milk, cultivated with primary antibodies to USP49 (18066‐1‐AP, Proteintech), p-PI3K (#4228, Cell Signaling Technology, Inc.), p-AKT (#4060, Cell Signaling Technology, Inc.), and GAPDH (#2118, Cell Signaling Technology, Inc.) overnight at 4 °C followed by another 1 h of incubation with secondary antibody horseradish peroxidase-conjugated goat anti-rabbit IgG. Finally, the blots were developed using a custom-made ECL detection system.

### Statistical analysis

Experimental data are expressed as the means ± standard deviation (s.d.) and analyzed using GraphPad Prism version 5.0 software (GraphPad Software, San Diego, CA, USA). Each experiment was repeated five times in the present study. Statistical differences between two groups were assessed using Student’s *t*-test and Multiple sets of comparisons were analyzed using one-way ANOVA. Statistical significance is indicated by *P* values <0.05. The correlation between the expression of miR-5000-3p and its target genes was determined by Spearman correlation analysis.

## Results

### Bioinformatics analysis reveals miR-5000-3p as a tumor promoter and contributes to OXA chemoresistance in CRC

**Fig. 1:**
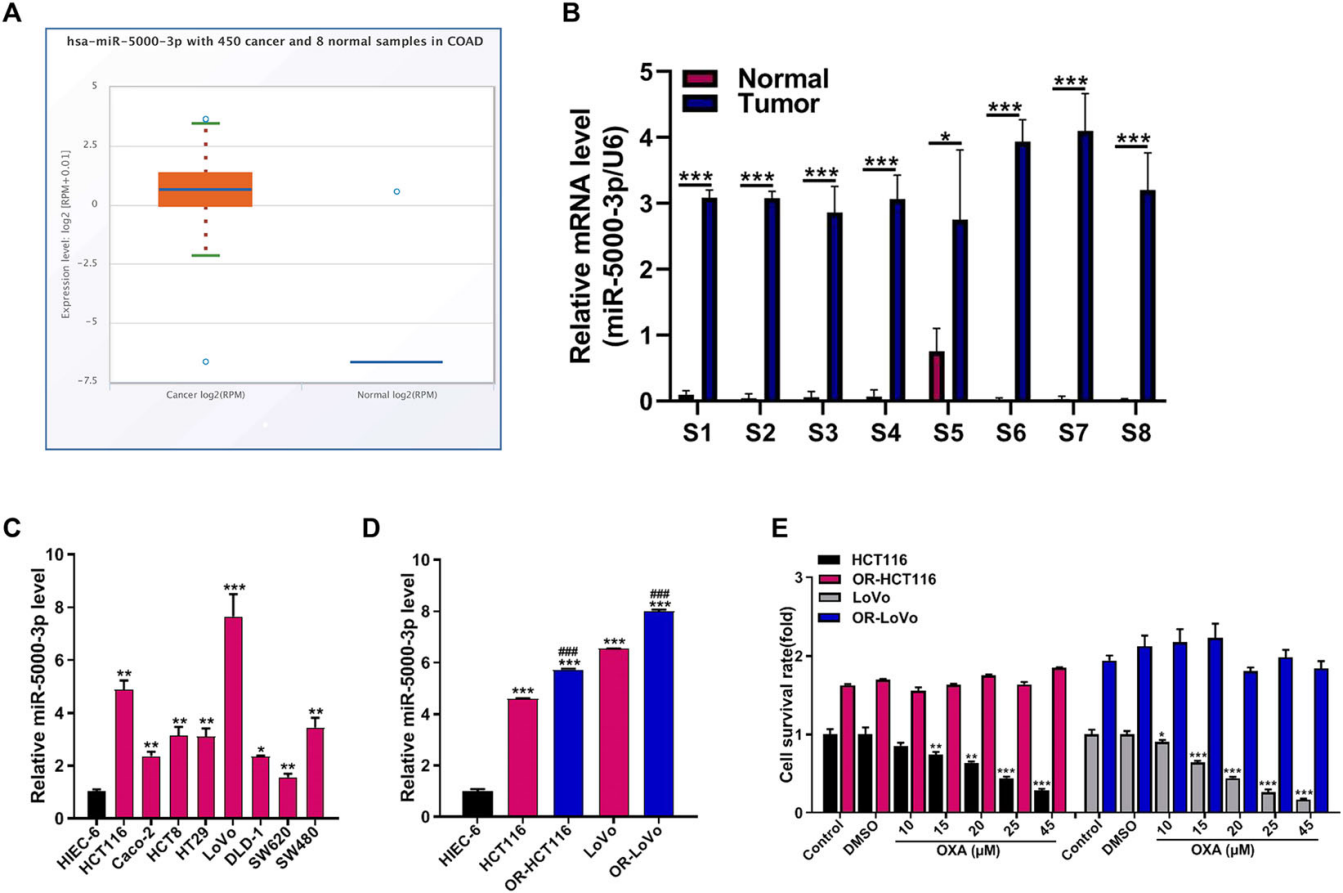
miR-5000-3p is highly expressed in CRC patients and cells. **A** The expression levels of miR-5000-3p in normal or CRC tissues from Colon Adenocarcinoma (COAD) were analyzed by starBase online, which was based on the TCGA database (http://starbase.sysu.edu.cn/). **B** Eight pairs of fresh primary CRC tissues and individual normal para-cancerous tissues were prepared for qRT-PCR against miR-5000-3p. U6 was used as an internal control. **C** The expression levels of miR-5000-3p in 8 CRC cell lines or normal control cell line from qRT-PCR assay. **D** The expression levels of miR-5000-3p in two CRC cell lines (HCT116 and LoVo) or their oxaliplatin (OXA)-resistant cell lines (OR-HCT116 and OR-LoVo) from qRT-PCR assay. **E** The MTT assay result indicates the survival rate of OR-HCT116 and OR-LoVo cells after treatment with or without OXA (45 µM at 24 h) compared with the HCT116 and LoVo cell control group. *p < 0.05, **p < 0.01, and ***p < 0.001; ^###^*p* < 0.001.

To identify that miR-5000-3p plays critical roles in CRC, we first analyzed the expression data in COAD of starBase v3.0 project. We found miR-5000-3p was overexpressed in 450 cases of COAD when compared with 8 normal samples from TCGA dataset (Fig. 1A). In addition, we chosen 8 CRC patients and investigated the expression of miR-5000-3p, then the qRT-PCR results showed that miR-5000-3p expression was also increased in all the 8 cases of CRC tissues compared with the paired adjacent normal tissues (Fig. 1B). Meanwhile, miR-5000-3p expression in CRC was associated with age (*p* = 0.08) and T Infiltrate (T1–T4) (*p* = 0.023) of 403 cases of CRC patients in Table 1. These results showed that miR-5000-3p as a tumor promoter in CRC.

### miR-5000-3p expression in CRC cell lines and its effect in chemoresistance

First, we examined miR-5000-3p expression in a panel of CRC cell lines and found that miR-5000-3p was remarkably increased in eight CRC cell lines compared with the immortalized human normal intestinal epithelial cell line HIEC-6 (Fig. 1C). To determine the effect of miR-5000-3p in chemoresistance, we employed the OXA-resistant CRC cell lines established in this study. Then, we also confirmed that miR-5000-3p expression differ between two CRC cells and two OR-CRC cells. RT-qPCR validated the upregulation of miR-5000-3p in OR-HCT116 and OR-LoVo cells compared with that in HCT116 and LoVo cells (Fig. 1D). As shown in Fig. 1E, the IC_50_ in HCT116 and LoVo cells was 15 µM, whereas IC_50_ in OR-HCT116 and OR-LoVo cells was 3-fold (45 µM) that of parental cells. MTT assay result indicates that the cell viability of OR-HCT116 and OR-LoVo cells was higher than that of HCT116 and LoVo cells for each control group (Fig. 1E). These data confirm the resistance of OR-HCT116 and OR-LoVo cells, which can resist up to 45 µM OXA treatment. These results also demonstrated that miR-5000-3p may play an important role in CRC cell resistance to OXA.

### miR-5000-3p regulates OXA chemoresistance, cell migration, invasion, and cell apoptosis in CRC and OR-CRC cells in vitro

**Fig. 2:**
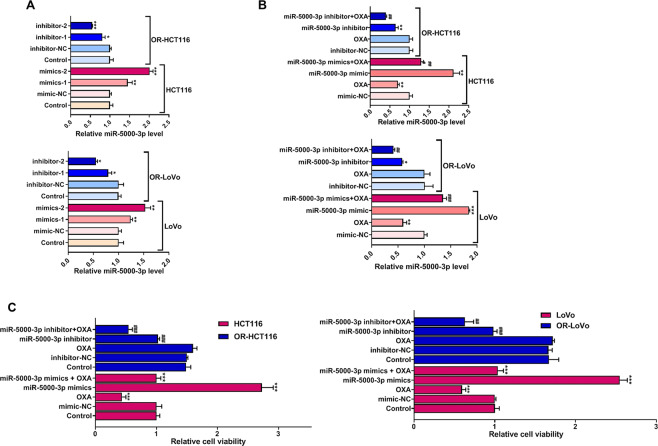
The effect of the overexpression or knockdown of miR-5000-3p and treatment with or without OXA on cell survival of HCT116 and LoVo or OR-HCT116 and OR-LoVo cells in vitro. **A** miR-5000-3p expression after transfections with miR-5000-3p inhibitors or mimics in two OR-CRC cell lines or two CRC cell lines. **B** miR-5000-3p expression in HCT116 and LoVo or OR-HCT116 and OR-LoVo cells transfected with a miR-5000-3p mimics (40 nM), inhibitor (40 nM), or NC miRNA (40 nM) (mimics negative control (NC) or inhibitor NC) or treatment with OXA (45 µ M). miR-5000-3p fold increase which was calculated by the ratio miR-5000-3p/U6, being U6 snRNA constitutively expressed. **C** Relative cell survival fold changed of HCT116 and LoVo cells or OR-HCT116 and OR-LoVo cells determined by the MTT assay. *p < 0.05, **p < 0.01, and ***p < 0.001; ^#^*p* < 0.05, ^##^*p* < 0.01, and ^###^*p* < 0.001.

To further confirm the role of miR-5000-3p in OXA chemoresistance of CRC cells, we transfected OR-HCT116 and OR-LoVo cells with miR-5000-3p inhibitors, and their respective parental cell lines (HCT116 and LoVo) were transfected with miR-5000-3p mimics vectors. RT-qPCR verified that miR-5000-3p inhibitor-2 and miR-5000-3p mimics-2 own the relative higher transfection efficiency to knockdown or upregulate miR-5000-3p expression, respectively (Fig. 2A). Therefore, miR-5000-3p inhibitor-2 and miR-5000-3p mimics-2 were chosen for subsequent experiments. Furthermore, Fig. 2B showed that the OXA treatment suppressed the expression of miR-5000-3p but did not eliminate the transfection ability of the miR-5000-3p mimics in HCT116 and LoVo cells. The miR-5000-3p inhibitor-2 was able to successfully suppress miR-5000-3p expression in OXA-treated OR-CRC cells (Fig. 2B). However, the expression of miR-5000-3p in two OR-CRC cells did not decrease when treated with OXA only, which reflected the successful establishment of OXA-resistant cell lines.

**Fig. 3:**
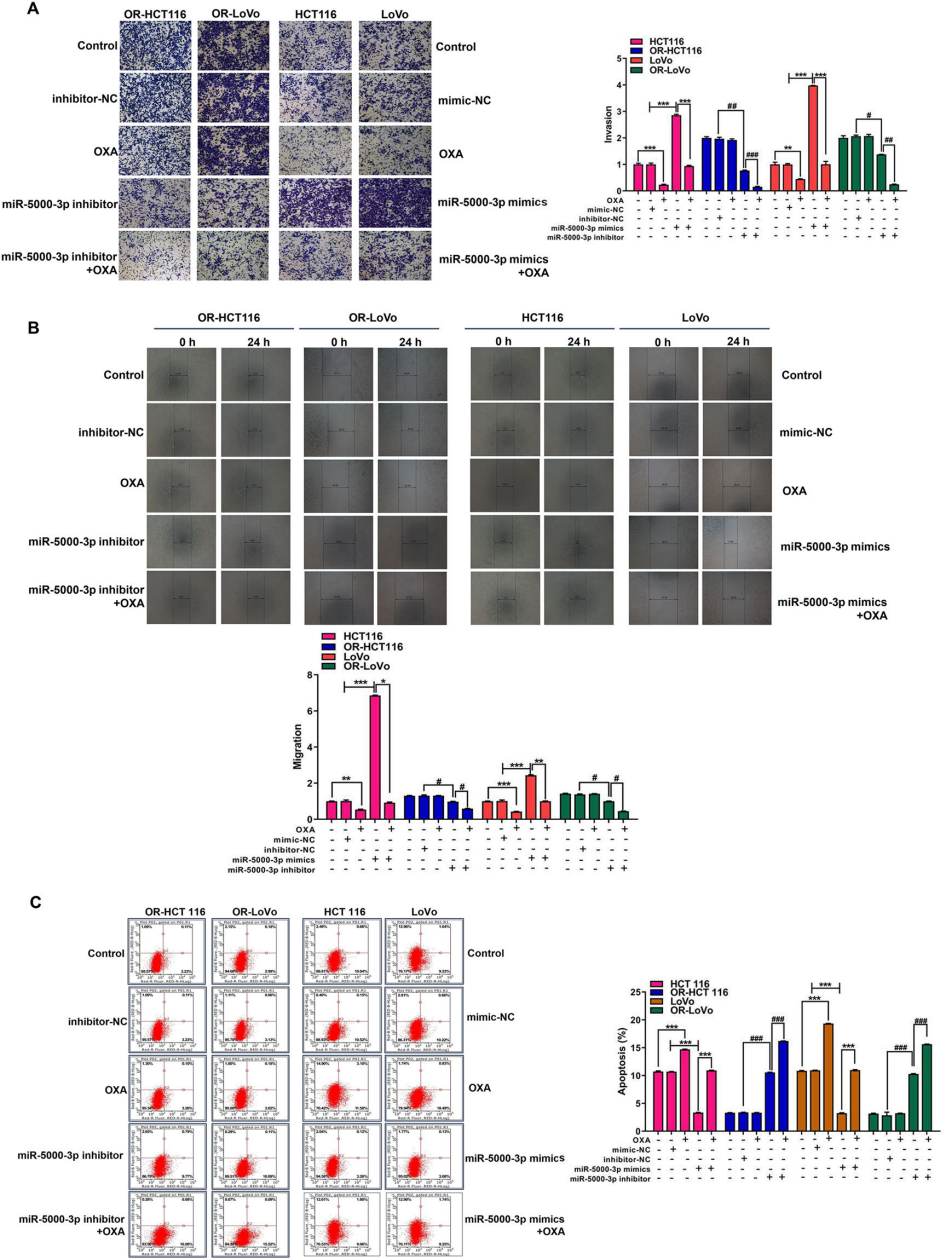
The effect of the overexpression or knockdown of miR-5000-3p and treatment with or without OXA on cell invasion, migration, and apoptosis of HCT116 and LoVo or OR-HCT116 and OR-LoVo cells in vitro. **A** Representative images and bar graphs showing the effects of miR-5000-3p overexpression or knockdown on cell invasion of HCT116 and LoVo or OR-HCT116 and OR-LoVo cells. **B** Effects of miR-5000-3p overexpression or knockdown on cell invasion of HCT116 and LoVo or OR-HCT116 and OR-LoVo cells were measured by wound-healing assays. **C** Effects of miR-5000-3p overexpression or knockdown on cell apoptosis of HCT116 and LoVo or OR-HCT116 and OR-LoVo cells were measured by flow cytometer assays. *p < 0.05, **p < 0.01, and ***p < 0.001; ^#^*p* < 0.05, ^##^*p* < 0.01, and ^###^*p* < 0.001.

Next, MTT assay demonstrated that two OR-CRC cells had a higher cell viability than two CRC cells, and two OR-CRC cells were both resistant to OXA treatment. Cell viability was suppressed after the knockdown of miR-5000-3p by transfecting OR-CRC cells with miR-5000-3p inhibitor; and more importantly, knockdown of miR-5000-3p enhanced the chemosensitivity of OR-HCT116 and OR-LoVo cells to OXA treatment (Fig. 2C). In contrast, overexpression of miR-5000-3p induced an increase in cell viability in HCT116 and LoVo cells after treatment with OXA when compared with OXA treatment only group (Fig. 2C). Moreover, we found that OXA decreased cell migration and invasion, and induced cell apoptosis in CRC cells but not in OR-CRC cells (Fig. 3A–C). Interestingly, miR-5000-3p mimics promote the capacities of migration and invasion, and suppressed cell apoptosis in HCT116 and LoVo cells after treatment with OXA. Separately, cell migration and invasion were reduced by miR-5000-3p inhibitor in both OR-HCT116 and OR-LoVo cells (Fig. 3A, B). Expectedly, Flow cytometry analysis showed that apoptotic cells were significantly increased in OR-CRC cells after treatment with miR-5000-3p inhibitor when compared with the OXA group; whereas overexpression of miR-5000-3p greatly inhibited the apoptosis of HCT116 and LoVo cells in the OXA group (Fig. 3C). Our findings revealed that miR-5000-3p played an important role in CRC cell resistance to OXA by promoting cell growth, metastases, and suppressing cell apoptosis.

### miR-5000-3p regulates tumor growth and chemosensitivity by targeting MIR22HG in a xenograft tumor model

**Fig. 4:**
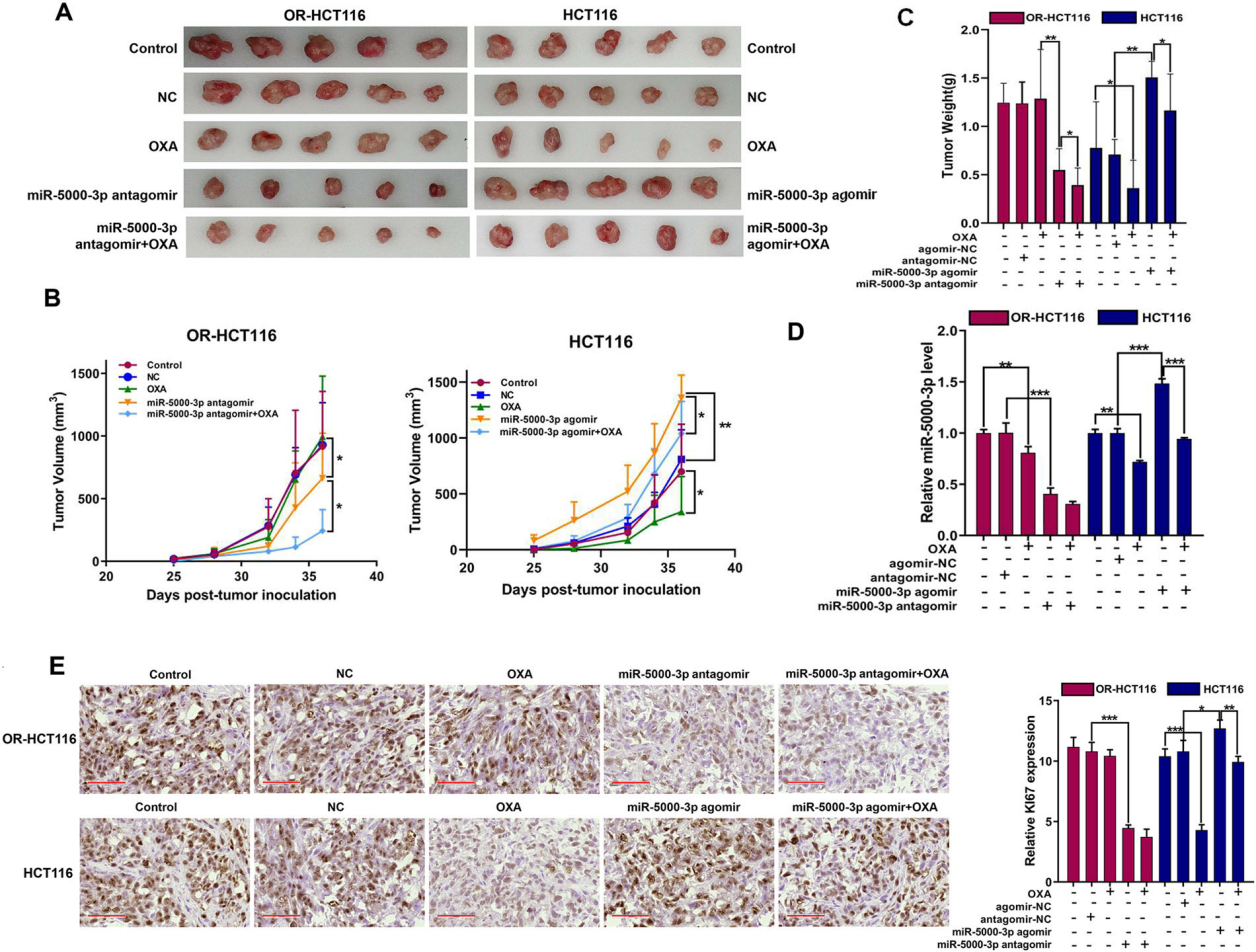
miR-5000-3p regulates cell proliferation and tumor growth. **A**–**C** HCT116 and OR-HCT116 cells were subcutaneously injected into nude mice. Tumor volumes (**B**) were measured at indicated days. Mice were sacrificed after 5 weeks. Tumor images were acquired as shown in (**A**) and tumor weights were measured as shown in (**C**). *n* = 5. **D** The mRNAs were extracted from the cells for miR-5000-3p detection and subjected to qRT-PCR. **E** Representative images and histogram of data statistics of immunohistochemical (IHC) staining of KI-67 in tumor tissues among different groups. Scale bar = 50 µm. *p < 0.05, **p < 0.01, and ***p < 0.001.

To assess whether miR-5000-3p exerts OXA-resistance function in vivo, we established the xenograft mouse models. When tumor growth reached 100 mm^3^, mice were treated with OXA, miR-5000-3p antagomir, miR-5000-3p agomir, or the combinations, As shown in Fig. 4A–C, the weight and tumor volume of mice injected with OR-HCT116 cells in control and NC groups increase over time, and the mice underwent significant weight loss in the miR-5000-3p antagomir group. Interestingly, only OXA treatment had little effect on tumor growth, but when miR-5000-3p was suppressed, OXA had a significant inhibition effect on tumor growth (Fig. 4A–C). Rather, we found that treatment with OXA significantly inhibited tumor growth of mice injected with HCT116 cells, but the miR-5000-3p agomir promoted tumor growth. Moreover, after tumors were injected with the miR-5000-3p agomir, tumor sensitivity to OXA was enhanced (Fig. 4A–C). These data suggest that miR-5000-3p induces tumor proliferation and modulates sensitivity to OXA drug chemotherapy in vivo.

We used qRT-PCR to analyze miR-5000-3p expression after treatment with OXA, miR-5000-3p antagomir, or m R-5000-3p agomir in two different tumors (Fig. 4D). We further analyzed tumor proliferation by detecting Ki-67 using IHC staining. The Ki-67 protein level in the OR-HCT116 control group was higher than that in the HCT116 control group. Treatment with agomir to upregulate miR-5000-3p induced Ki-67 upregulation in parental HCT116 cells, and knockdown of miR-5000-3p in OR-HCT116 cells decreased the Ki-67 protein level when compared with that in the control and NC groups (Fig. 4E). Importantly, the overexpression of miR-5000-3p decreased OXA-induced injury in HCT116 tumors, and the knockdown of miR-5000-3p expression enhanced sensitivity to OXA in OR-HCT116 cells. Accordingly, our results in vitro and in vivo indicate that OR-HCT116 cells are resistant to OXA as a result of increasing the expression of miR-5000-3p, which induces tumor growth and proliferation.

### USP49 is a downstream target of miR-5000-3p in CRC cells

**Fig. 5:**
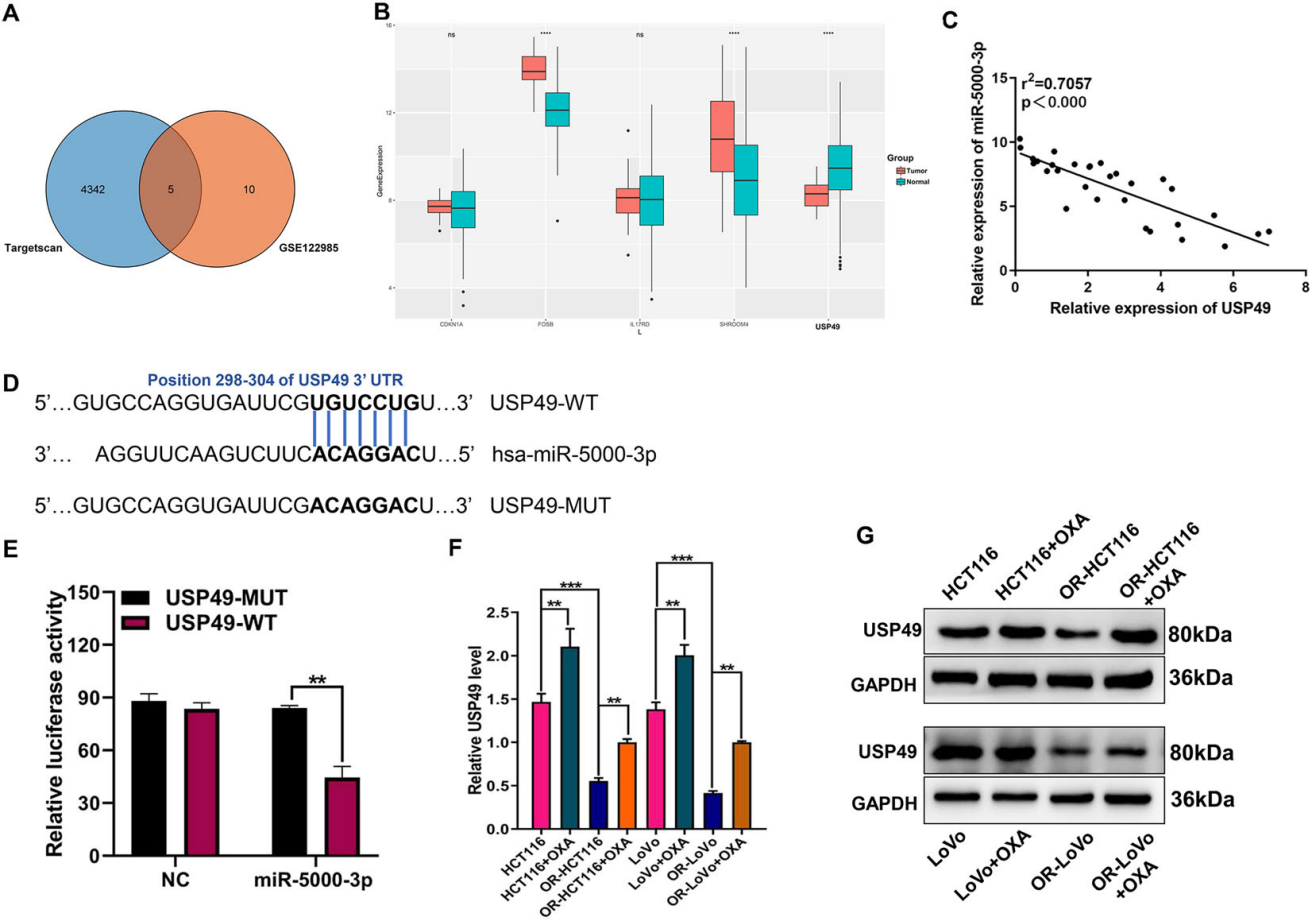
miR-5000-3p regulates USP49 mRNA and protein expression by targeting the 3′UTR in OR-CRC cells. **A** We combined the TargetScanHuman 7.2 databases predicting the putative targets of miR-5000-3p and GEO dataset screening DEGs between CRC cells and OR-CRC cells, and there were five predicted targets that overlapped among the two online databases. Venn diagrams showing the five DEGs (*p* < 0.05 & |logFC|>1). FC, fold change. **B** The expression levels of CDKN1A, SHROOM4, FOSB, USP49, and IL17RD in normal or CRC tissues from Colon Adenocarcinoma (COAD) were analyzed by starBase online, which was based on the TCGA database (http://starbase.sysu.edu.cn). **C** The scatter plots showing correlation between expression of miR-5000-3p and USP49 in COAD. *r*^2^ = 0.7057 and *p* < 0.000. **D**, **E** Luciferase activity assays of the activity of luciferase vectors containing the USP49 3′UTR were performed following transfection with miR-5000-3p or negative control (NC) for 24 h using the Dual-Luciferase Reporter Assay System. **F**, **G** The mRNA and protein expression levels of USP49 in two CRC cell lines (HCT116 and LoVo) or their oxaliplatin (OXA)-resistant cell lines (OR-HCT116 and OR-LoVo) or after OXA treatment from qRT-PCR assay (**F**) and western blot (**G**). *p < 0.05, **p < 0.01, and ***p < 0.001.

**Fig. 6:**
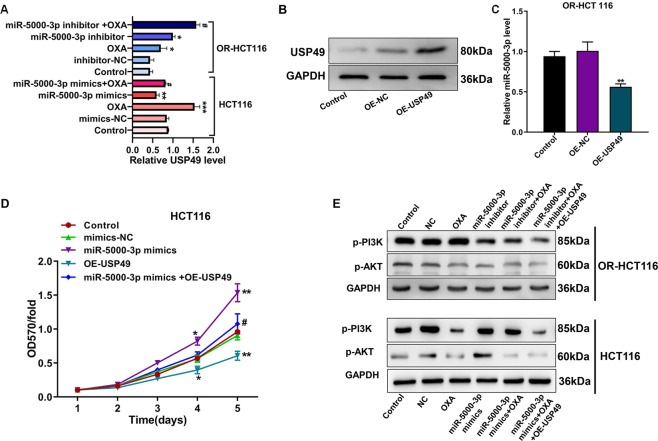
miR-5000-3p regulates PI3K/AKT signaling pathway by targeting USP49 in CRC or OR-CRC cells. **A** Relative mRNA level of USP49 in HCT116 or OR-HCT116 cells transfected with a miR-5000-3p mimics, or inhibitor, or treatment with OXA. **B** Protein expression of USP49 in OR-HCT116 cells after transfected with HSP49 overexpression or NC vectors were detected by western blot. **C** miR-5000-3p expression after transfections with USP49 overexpression or NC vectors in OR-HCT116 cells. **D** MTT assay was used to detect cell proliferation viability in different groups following 5 days. **E** Western blot showing the protein level of PI3K/AKT signaling relevant markers, includes p-PI3K and p-AKT. *p < 0.05, **p < 0.01, and ***p < 0.001 vs Control or NC; ^#^*p* < 0.05 vs inhibitor or mimics.

As shown in Fig. 5A, we determined 15 different expressed genes (DEGs) between TargetScan dataset and GEO dataset (GSE122985: *p* < 0.05 & |logFC|>1). With the aim of finding the specific target gene of miR-5000-3p in CRC, we used TargetScanHuman 7.2 to predict the potential mRNAs that have the capacity of binding with miR-5000-3p, and 4347 potential target genes were identified under the screening condition. Then genes from both approaches (DEGs from GEO and target genes predicted) were overlapped and 5 putative targets were selected for further functional analyses. In order to explore the expression of these five genes in a larger number of samples and strengthen the reliability of our results, the authors subsequently searched and downloaded TCGA data for further analysis. The results demonstrated that level of USP49 was reduced markedly in CRC tissues, while IL17RD, FOSB, CDKN1A, and SHROOM4 were obviously increased in CRC tissues, when compared to that in non-cancerous tissues (Fig. 5B). Another, according to the Context++ score percentile (score > 70) in the TargetScanHuman, we selected the tumor suppressor USP49 with high Context++ score (75) for the subsequent analysis when compared with IL17RD (score = 58), FOSB (score = 88) and SHROOM4 (score = 21). Interestingly, there was a significantly negative correlation between USP49 and miR-5000-3p in CRC (Fig. 5C). USP49 is one of the target genes for miR-5000-3p in our study and Fig. 5D displays a binding site between USP49 and miR-5000-3p obtained from TargetScanHuman 7.2. Subsequently, based on the results of a luciferase reporter assay, we discovered that overexpression of miR-5000-3p reduced the luciferase activity of WT-USP49 3′-UTR constructs but had no obvious effects on the luciferase activity of Mut-USP49 3′-UTR constructs (Fig. 5E). Moreover, USP49 mRNA and protein expression in OR-CRC cells were both lower compared with two CRC cell lines (Figs. 5F and G). Treatment of CRC cells and OR-CRC cells with OXA increased the USP49 mRNA and protein expression when compared with that in the untreated cells (Figs. 5F and G). As presented in Fig. 6A, the overexpression of miR-5000-3p reduced USP49 level in HCT116 cells while knockdown of miR-5000-3p elevated USP49 expression in OR-CRC cells, respectively. The expression of USP49 was increased following OXA treatment in OR-CRC cells transfected with miR-5000-3p inhibitor (Fig. 6A). These data suggest that miR-5000-3p is a downstream gene of USP49.

### USP49 suppresses miR-5000-3p expression and regulates PI3K/AKT signal pathway

Accordingly, we aimed to confirm that USP49 is a direct target gene of miR-5000-3p. Then, the USP49 expression in OR-HCT116 cells could be obviously upregulated using overexpression transfection (Fig. 6B). The qRT-PCR data show that the expression of miR-5000-3p decreased in OR-HCT116 cells expressing high USP49 compared with that in control and OE-NC cells (Fig. 6C). In order to investigate the effect of USP49 on miR-5000-3p in CRC, we transfected OE-USP49 plasmid into the miR-5000-3p overexpression HCT116 cells, and then MTT was performed to detect cell proliferation ability. As shown in Fig. 6D, overexpression of USP49 significantly inhibited cell proliferation and reversed the promotion effect of miR-5000-3p on cell proliferation ability.

Due to USP49 was involved in the PI3K/AKT signaling pathway^10^. Western blot results demonstrated that miR-5000-3p inhibitor could suppress PI3K/AKT signaling pathway reducing the expression of p-PI3K and p-AKT at protein levels in OR-CRC cells while miR-5000-3p mimics promoted PI3K/AKT signaling (Fig. 6E). Treatment with OXA alone could strikingly suppress PI3K/AKT signal in CRC cells but not in OR-CRC cells. Interestingly, following overexpression or knockdown of miR-5000-3p separately being transfected into the parental cells or resistant cells, OXA treatment all could restrain PI3K/AKT signaling pathway by decreasing p-PI3K and p-AKT at the protein levels (Fig. 6E). This inhibitory effect on PI3K/AKT signaling can be compounded by overexpression of USP49 (Fig. 6E). These findings demonstrated that knockdown of miR-5000-3p expression enhanced the sensitivity to OXA in resistant CRC cells binding to USP49 through regulating PI3K/AKT signaling pathway.

## Discussion

Because of the lack of an early and effective diagnosis biomarker, the majority of the CRC patients were diagnosed with advanced-stage metastasis, leading to a disappointing survival rate. Increasing studies have revealed the significant function of miRNAs on the complicated courses involved in the biological progressions of colorectal cancer, and miRNAs negatively and post-transcriptionally regulate gene expression and function as oncogenes or tumor suppressors. For instance, miRNA-144, as a suppressor factor, could suppress proliferation and migration of CRC HCT116 cells^14^. Due to resistance to OXA based regimens^15,16^, low miRNA-148a and miRNA-625-3p expression contribute to tumor budding, which is thought to represent epithelial–mesenchymal transition in CRC^17^. miR-31-5p not only plays an important role in the development of CRC but also ultimately induces tumor growth and resistance in cancer cells by directly targeting LATS2^13^. Only a few recent researches have revealed the carcinogenesis of miR-5000-3p in cancers, such as laryngocarcinoma and CRC^3,7^. However, the underlying mechanisms of miR-5000-3p in CRC remain to be detected. In the present study, we determined the expression pattern and functions of miR-5000-3p and provided several evidence indicating that miR-5000-3p acts as a tumor promoter in CRC. We found miR-5000-3p was increased in clinical samples and most CRC cell lines. Importantly, high level of miR-5000-3p in CRC patients was associated with high age and T Infiltrate and predicted poor prognosis in patients with CRC, as well as miR-5000-3p expression was also found to be increased in the oxaliplatin-resistant (OR)-CRC cells.

Drug resistance is a major problem in cancer chemotherapy. Furthermore, miR-5000-3p downregulation exerted repressive effects on cell proliferation and migration while promoted cell apoptosis and the sensitivity to OXA in OR-CRC cells. Besides, we also found that upregulation of miR-5000-3p promoted proliferation and migration while suppressed cell apoptosis and the cytotoxicity of OXA in CRC cells. In addition, our findings demonstrated that tumor weight and size were significantly reduced after inhibiting miR-5000-3p in the xenograft tumor models which were injected with OR-HCT116 cells while only OXA treatment has no significant effect. This evidence suggests that the function of miR-5000-3p in human colorectal tumorigenesis and drug resistance may offer a new strategy for future colorectal cancer therapy. We also found that the capacities of proliferation of tumors in vivo were inhibited by miR-5000-3p inhibitor. Accordingly, we speculated that miR-5000-3p plays an important role in the development of CRC and ultimately induces tumor growth and resistance in cancer cells.

A failure in regulating proliferation together with suppression of apoptosis are the minimal requirements for a cell to become cancerous^18^. A number of these studies have already been discovered in the literature, that USP49 was downregulated in several cancer as a tumor suppressor. For instance, USP49 was lowly expressed in NSCLC, and patients with high expression of USP49 possessed a relatively long overall survival^10^. In addition, overexpression of USP49 sensitized cancer cells to chemotherapy^9^. In the present study, low expression of USP49 was observed in CRC patients and CRC cells, and drug-resistance cells own a lower expression of USP49 comparing to the parental cells. miRNAs negatively regulate the expression of target genes by binding to 3′UTR segments of messenger RNAs and are aberrantly expressed in most human cancers, including CRC, in which they may function as tumor suppressors or as oncogenes^19^. In our study, miR-5000-3p directly targeted USP49. Clinical studies have also found that the decreased expression of low USP49 expression in CRC patients and closely related to a poor prognosis of CRC patients. In the OXA-resistance CRC cells, miR-5000-3p inhibitor obviously stimulated USP49 expression, which is consistent with the result of USP49 overexpression suppressed miR-5000-3p level, suggesting there is a negative relationship between miR-5000-3p and USP49 in CRC. Accumulating evidence indicates that abundant of USP49 showed some restrain on tumorigenesis and proliferation by inhibiting PI3K/AKT pathway in several cancers^9,10^. Thus, we found downregulation of miR-5000-3p and USP49 overexpression all could restrain PI3K/AKT pathway signaling pathway in OR-CRC cells. In line with this, we reveal a mechanism by which USP49 being regulated by miR-5000-3p, is related to chemoresistance and promotes tumor progression via the PI3K/AKT pathway in CRC.

In summary, the expression of USP49 was altered by miR-5000-3p, indicating that they are functionally related. We demonstrated a novel chemoresistance mechanism in colorectal cancer by performing in vitro and in vivo experiments that regulated the expression of USP49, which was found to be a transcription factor of miR-5000-3p. We speculated that knockdown of miR-5000-3p reduced tumor growth and chemoresistance in both cancer cells and tumors. These results suggest that miR-5000-3p may be a novel biomarker for detecting the degree of colorectal cancer malignancy. Moreover, miR-5000-3p and its targeting genes can be considered as new therapy targets in cancer patients who develop resistance to chemo drugs in clinical trials.

## Data Availability

The authors declare that all data supporting the findings of this study are available within the article or from the corresponding author upon reasonable request.
